# A cerebrovascular event turning pericarditis into vasculitis

**DOI:** 10.1007/s12471-025-01942-7

**Published:** 2025-04-28

**Authors:** Ines Frederix, Madelon van den Heuvel

**Affiliations:** Department of Cardiology, Zuyderland Hospital, Heerlen, The Netherlands

**Fig. 1:**
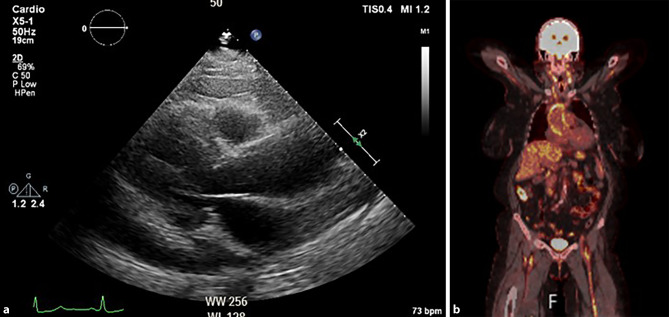
**a**. Transthoracic echocardiography showing diffuse pericardial effusion. **b**. PET-CT scan illustrating thickened and metabolically active walls of the large vessels

A 64-year-old woman presented with fatigue, night sweats, and dyspnoea. Physical examination was normal. The electrocardiogram showed no abnormalities. Blood testing revealed a CRP of 185 mg/l and a troponin level of 20 ng/L. An echocardiography disclosed a diffuse pericardial effusion of 17 mm (Fig. 1a). She received colchicine and carbasalaat calcium for a suspected pericarditis.

One day later she developed right quadrantanopsia. CT angiography revealed an occlusion in P4 of the left arteria cerebri posterior. Additional testing showed an ESR of 115 mm/hour. PET-CT showed metabolically active walls of the large vessels, compatible with vasculitis (Fig. 1b). Prednisolone was started.

After 4 weeks she had regained her vision. The pericardial effusion had decreased to 5 mm, CRP was normalized, and the ESR dropped to 64 mm/hour. This case highlights the importance of recognising pericarditis as the first manifestation of large vessel vasculitis [1–5].
